# Bee positive: the importance of electroreception in pollinator cognitive ecology

**DOI:** 10.3389/fpsyg.2013.00445

**Published:** 2013-07-17

**Authors:** Mathieu Lihoreau, Nigel E. Raine

**Affiliations:** ^1^School of Biological Sciences and the Charles Perkins Centre, The University of SydneySydney, NSW, Australia; ^2^School of Biological Sciences, Royal Holloway University of LondonEgham, Surrey, UK

The global atmospheric circuit generates a permanent electric field between the Earth surface and outer atmosphere (Rycroft et al., 2000). The ground and plants conductively linked to it are negatively charged (Bowker and Crenshaw, 2007), whereas animals build up positive charge as they move in contact with air molecules (Jackson and McGonigle, 2005). Electric fields emanating from plants and pollinators, such as bees, are believed to promote pollination by enabling pollen grains to “jump” from flowers to pollinators and vice versa (Corbet et al., 1982). Two recent studies reveal that bees not only detect these electric fields but also learn to discriminate them, indicating that electroreception should be seriously considered alongside vision and olfaction when studying bee behavior and ecology.

Writing in *Science*, Clarke et al. (2013) demonstrated that bumblebees (*Bombus terrestris*) detect electric fields around plants and learn to use them to decide whether or not to visit flowers. Using a Faraday pail to measure electric fields generated by bees and plants, the team described how a bee visit temporarily modifies the electric charge of (Petunia) flowers, suggesting that floral electric properties could be used by future visitors to assess the reward value without necessarily needing to probe the flower.

To explore this possibility, the authors used differential conditioning in which bees were trained to associate an electrically charged feeder (30 V) with a sucrose reward (CS+) and an uncharged feeder with an aversive quinine solution (CS−). After extensive training (50 trials), bees chose the rewarding feeder in around 80% of trials. Similar levels of performance were observed when bees were trained with two feeders carrying the same charge but different electric field patterns (homogeneous vs. bull's eye shape), indicating that these insects can learn both the magnitude and geometry of an electric field. Bees learned to perform even better in discrimination tasks if the two feeders differed both in color (shade of green) and their electric field pattern compared to if they differed only in color. Natural electric fields around flowers may therefore contribute to the multimodal sources of information that bees use to learn and memorize floral rewards, in conjunction with color, pattern, shape, texture, humidity, or warmth (e.g., Stach et al., 2004; Dyer et al., 2006; Raine and Chittka, 2008).

In another study published in the *Proceedings of the Royal Society of London*, Greggers et al. (2013) went a step further asking both about the mechanisms of electroreception and their potential role in communication. They measured the electric fields emanating from honeybees (*Apis mellifera*) in various contexts, including during the “waggle dance”: the figure-of-eight-shaped circuit performed by foragers upon their return to the nest to communicate the location of rewarding flowers to nestmates (Von Frisch, 1967). Comparison of electrical recordings to video clips of dancing bees revealed that the sequence of wing beats and abdomen movements performed by dancers generates a specific modulated electric field composed of pulses of several hundred volts. To test whether bees can detect and discriminate such electric cues, the team stimulated tethered bees with either constant or modulated electric fields. Stimuli mimicking those of a waggle dancer (among others) triggered both antennal movements and walking activity, suggesting that electric cues from conspecifics evoke behavioral responses in a social context.

To identify the sensory receptors involved in electroreception the authors used proboscis extension response conditioning, a classical paradigm to study olfactory learning and memory (Tadeka, 1961; Menzel, 2012). Harnessed honeybees were trained to associate an electrically charged stimulus (CS+) with a sucrose reward while an uncharged stimulus (CS−) was not rewarded. Whilst intact bees rapidly learned to discriminate the electrical stimulus, those whose antennae had either been removed or entirely coated with wax (to prevent movement and block sensory receptors) were unable to learn this cue. Interestingly, if only the antennal articulations were free of wax, bees could detect vibrations and were able to discriminate and learn electrical cues to some extent. Electrophysiological recordings of the mechanosensory neurons in the Johnston's organ, a structure located in the second antennal segment involved in detecting acoustic vibrations (Ai, 2010), confirmed that electric stimulation induces neuronal responses. Therefore, much in the same way as body hairs are thought to contribute to human electric field perception (Chapman et al., 2005), passive antennal deflection caused by electrical stimulation may activate mechanosensory organs responsible for electroreception in bees.

The observation that insects can sense electric fields is not new. Indeed, it has long been known that electric fields induce a range of behavioral responses in ants, cockroaches, mosquitoes, fruit flies, and bees [reviewed in Jackson et al. (2011)]. However, previous studies have typically focused on electric fields orders of magnitude stronger than those usually found in nature, such as those generated by high voltage power lines (Greenberg et al., 1981; Newland et al., 2008). By questioning the biological relevance of insect electroreception using ecologically realistic electric cues, these recent observations on bees open a whole new window into the study of insect sensory, cognitive, and behavioral ecology. Although neither of these laboratory studies yet provide a clear answer about how bees actually detect and process electric fields, or indeed whether electrical information is really used by bees under field conditions, they suggest that electroreception could be particularly important in a foraging context: both to assess the pollination status of flowers and to communicate their locations. Like many nectarivores, bees face complex routing problems to efficiently visit continuously replenishing and patchily distributed resources (Lihoreau et al., 2010, 2012). Electric fields naturally emanating from important features of their environment, such as the nest (hive), flower patches, bushes, or treelines, could provide relevant information in addition to visual cues (Chittka et al., 1995), polarized light (Wellington, 1974), or magnetic fields (Collett and Baron, 1994) to assist accurate navigation and route finding. Experimental manipulations of local electric fields in bees' foraging areas could test this hypothesis and help to clarify the importance of “electrical cognition” in these animals.
